# Developing quality indicators for the care of patients with musculoskeletal injuries in the Emergency Department: study protocol

**DOI:** 10.1186/s12873-017-0124-7

**Published:** 2017-05-05

**Authors:** Kirsten Strudwick, Anthony Bell, Trevor Russell, Melinda Martin-Khan

**Affiliations:** 1 Emergency and Physiotherapy Departments, QEII Jubilee Hospital, Metro South Hospital and Health Service, Brisbane, Queensland Australia; 2 School of Health and Rehabilitation, The University of Queensland, Brisbane, Queensland Australia; 3 Department of Emergency Medicine, The Royal Brisbane and Women’s Hospital, Metro North Hospital and Health Service, Brisbane, Queensland Australia; 4 School of Medicine, The University of Queensland, Brisbane, Queensland Australia; 5 Centre for Research in Geriatric Medicine, School of Medicine, The University of Queensland, Brisbane, Queensland Australia; 6 Centre for Online Health, School of Medicine, The University of Queensland, Brisbane, Queensland Australia

**Keywords:** Emergency service, Quality indicators, Quality of health care, Wounds and injuries, Musculoskeletal diseases, Research design

## Abstract

**Background:**

Musculoskeletal injuries are a common presentation to the Emergency Department (ED). The quality of care provided is important to the patients, clinicians, organisations and purchasers of care. In the context of the increasing burden of musculoskeletal disease, quality of care needs to occur despite financial impacts, variations in care, and pressure to reach time-based performance measures. This study aims to develop a suite of evidence-based quality indicators (QI) which will provide a measure of the quality of care for patients with musculoskeletal injuries in the ED.

**Methods:**

This study will utilise a multi-phase mixed methods protocol, commencing with a systematic review of the literature to identify and critically appraise existing QIs for musculoskeletal injuries in the ED. The study will then build on the gaps identified in the review to develop a suite of preliminary QIs, in accordance with established research methodology under the governance of an expert panel. The developed QI set will then be field-tested for feasibility and validity in selected EDs. After field-testing, the suite will be refined in consultation with the expert panel and finalised using a formal voting process.

**Discussion:**

The assessment of performance against QIs provides a quantitative measure for the quality of care provided to patients, to identify and target quality improvement activities. The QIs developed through this study will be evidence-based and balanced across the areas of structures, processes and outcomes. The rigorous methodology used to develop and test the QIs will result in QIs that are meaningful, valid, feasible to collect and efficiently measurable, amenable to improvement, and selected by experts in the emergency medicine field. The final QI suite will have applications across EDs that affords comparison, benchmarking and optimisation of emergency care for patients.

## Background

The demand on Emergency Departments (EDs) is increasing each year [1–3], which can be attributed to population increases, an aging population, access block, a reduction in availability of bulk-billing general practitioners, and reduced operating hours of general practices [3–6]. Patients with musculoskeletal complaints constitute approximately 10–15% of all presentations to the ED [7, 8] and are increasingly using EDs as their primary mode of access to healthcare [9, 10]. Acute musculoskeletal injuries requiring ED services should be managed appropriately in this setting given that musculoskeletal conditions are a leading cause of disease burden world-wide [11–13].

Given the rising burden of musculoskeletal disease, improved quality of care for musculoskeletal injuries has become increasingly important to patients, clinicians, organisations, policy-makers and purchasers of care. Emergency medicine healthcare models and priorities vary around the world. For example, in the United States of America (USA), the focus in health care is on developing payment and delivery models that incentivise and support the provision of high quality, cost-efficient care [14]. Alternatively, the United Kingdom (UK) and Australasian systems focus on quantifying health care performance using time-based process measures that do not necessarily address patient outcomes [15–17]. These time-based performance measures, while effective at streamlining ED care, have not been universally associated with better outcomes for patients. A strong reliance on time-based performance measures does not necessarily correspond to high levels of quality across all aspects of ED care, and other important measures of quality, such as unplanned return visits and resource allocation to non-emergency activities, can be overlooked [18, 19].

While literature suggests certain time-based outcomes have improved with these time-performance targets [18, 20], few studies have explored the effects of such policies on the quality of ED care, particularly for conditions such as musculoskeletal injuries. In Australia, it is recommended that the ED four-hour length of stay target should be supported by a suite of associated indicators, with an emphasis on safety and quality, that measures aspects of the whole patient care process [21–23]. The addition of these secondary quality indicators (QIs) in the clinical management of patients has the potential to influence health outcomes [24] and enables benchmarking between facilities which can focus improvement efforts [25]. This is important considering the principle of value-based health care delivery centers on quality improvement, where quality is assessed as health outcomes [24].

It is recognised that health care delivery should be individualised and organised around medical conditions, rather than patients being broadly grouped together [24]. It follows that QIs should be focused on specific clinical conditions to make them more meaningful for quality assessment and improvement [26, 27]. The need for indicators which specifically measure musculoskeletal injuries is supported by The National Quality Forum (NQF) who launched a Musculoskeletal Measure project in 2013 in the USA, stating that “*because of the burden of musculoskeletal disease, there is a critical need for nationally recognised musculoskeletal care measures*” [28]. A recent consultation report involving emergency medicine stakeholders in Australia recommended that there is value in developing a suite of standardised, evidence-based clinical outcome indicators for specific conditions, and that this would likely be modelled on the Australian Council on Healthcare Standards accreditation process and based on current clinical audit processes [29]. While these would be based on Australian accreditation processes, they would likely have broad applicability in other jurisdictions also.

As contemporary ED care models evolve, there are now different personnel (e.g. nurse practitioners and advanced scope physiotherapists) assessing and managing ED patients with musculoskeletal injuries, in addition to medical officers. Current evidence highlights differences in the management, follow-up and patient satisfaction for this patient cohort between different ED personnel [30–37]. As such, a strategy that serves to codify and benchmark musculoskeletal care has the potential to guide improvements in quality, and reduce variations in care.

The intent of this multi-phase research project is to develop a clinical quality framework for EDs, enabling quality benchmarking for patients presenting with musculoskeletal injuries. The specific contribution that this research will make is to develop QIs that will drive change at a clinical level.

## Methods and design

### Study design

**Fig. 1:**
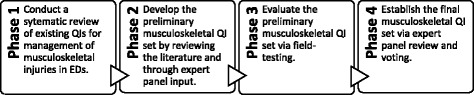
Flow chart of the study design

This protocol will use a four-phase mixed-methods study design (Fig. 1) which is modelled from an existing protocol for QI development [38, 39].

### Phase 1: systematic review of existing musculoskeletal QIs

*(This phase was completed in 2015).*

#### Aim

The aim of this phase is to identify existing musculoskeletal quality indicators developed for ED use, and to critically evaluate their methodological quality.

#### Design

Key databases should be searched, including MEDLINE, EMBASE, CINAHL and the grey literature, including relevant organisational websites, combining search terms of keywords and MeSH terms for emergency, quality of care and musculoskeletal injuries. English-language articles that describe the development of at least one QI related to the ED care of musculoskeletal injuries are to be included. Screening and application of inclusion/exclusion criteria by two authors is to be conducted for each article, according to the PRISMA checklist. [40] Data extraction and analysis of each included article involves extracting core data elements by one author against a standardised form, followed by a quality assessment by four authors, rating each relevant QI against the Appraisal of Indicators through Research and Evaluation (AIRE) Instrument [41]. QIs with similar definitions are to be grouped together and categorised according to the Donabedian [42] and the Institute of Medicine (IOM) [43] healthcare quality frameworks.

### Phase 2: develop the preliminary musculoskeletal QI set

*(This phase was completed in 2017).*

#### Aim

The aim of Phase 2 is to develop a preliminary QI set through evaluation of available scientific literature and expert panel input. Across the spectrum of commonly presenting musculoskeletal injuries in EDs, specific areas of interest include: history taking, physical assessment, different aspects of pain management, imaging and investigations, injury management, referral and follow-up pathways, patient safety, length of stay, adverse events, patient outcomes post-discharge from the ED, and patient satisfaction. The expert panel will nominate any additional topics believed to be of importance. The resultant preliminary QI set will aim to encompass structure, process and outcome domains, and will aim to be feasible for use in EDs across multiple hospitals.

#### Design – first expert panel meeting

The first expert panel will be guided by previous publications on the methodology of QI development [38, 39, 44], and the rapid review series outlined below. The face-to-face meeting will be held over 2 days, comprising 10–15 key stakeholders in the ED care of musculoskeletal injuries (Emergency Medicine Specialists, Orthopaedic Specialists, Emergency Physiotherapy Practitioners, Nurse Practitioners, quality improvement experts, and consumers). The meeting will commence with an overview of the study proposal, QI development methodology, and potential data collection tools for each QI. The panel will be lead through a formal process of review, including:

- A general discussion of the literature
- Review of existing QIs [45] with suggestions for modification if required
- Review of the list of potential QIs which are based on evaluations of the scientific literature
- An opportunity for the panel to recommend new QIs.

#### Design – rapid review series

The focus of the reviews will cover common musculoskeletal injuries of the foot and ankle, knee, lumbar spine, cervical spine, shoulder, elbow, wrist and hand that are commonly seen in the ED. These areas represent approximately 90% of all musculoskeletal and orthopaedic diagnostic areas as determined by the Emergency Department Information System (EDIS) for all EDs across Queensland for a one-year period in 2013–14.

Evaluation of the scientific literature will be undertaken to investigate the following:

1. Descriptors of best practice pertaining to musculoskeletal injury assessment and management within EDs, in terms of process and structure and the relationship of these to desired outcomes.
2. Musculoskeletal-specific quality emergency medicine management including feasibility of collecting the QIs, benchmarking in EDs, quality improvement projects, and identified barriers to achieving quality care.

The National Health and Medical Research Council (NHMRC) guidelines for systematic review of scientific literature will be adhered to [46]. Database searches will be conducted of PubMed, CINAHL, EMBASE and TRIP, combining keywords and MeSH terms for emergency medicine, best practice, and the body region or injury of interest. The grey literature will also be searched, including web-based literature and websites of organisations and societies pertaining to musculoskeletal injuries in EDs. One author will independently screen all articles, applying inclusion/exclusion criteria, adhering to the PRISMA checklist [40], followed by data extraction of core data elements against a standardised form by one of two authors. Each included article will be assigned a level of evidence per the NHMRC levels of evidence hierarchy [47].

The results from each included article will be grouped together into different categories across the clinical cycle of care, including assessment, diagnostic tests, pharmacological management, non-pharmacological management, and follow-up care categories, and will provide the evidence-base behind the potential QIs.

The data extraction tables, clinical cycle of care summaries, list of potential QIs and the list of existing QIs will be distributed to the expert panel for review prior to the meeting, and will be used to generate discussion at the expert panel meeting.

#### Data collection and compilation

The expert panel will provide their input via frank and open discussion. QIs will be redefined as required, and additional areas of quality of care will be identified if needed. Throughout the meeting, two scribes will record decisions and concepts discussed, and the meeting will be voice recorded to ensure all relevant discussion is captured. The expert panel members will be asked to give each indicator a tick for approval, or a cross for disapproval, next to each potential QI listed on their forms. This will be performed to gauge acceptability at this point and to support decisions around data collection. QIs that are given a cross for disapproval by all members of the panel will be excluded at this point, and QIs that are not feasible to collect will also be excluded, and noted for discussion at the next panel meeting. The expert panel will also be asked to document any other relevant points of discussion under each potential QI. These forms will be left with the research team at the conclusion of the meeting.

Following the first expert panel meeting, data collection tools will be developed to score the preliminary QIs. The QIs will be incorporated into a working manual for each indicator set (i.e. structure, process, outcome). Each QI will include detailed data specifications, such as numerator, denominator, exclusion characteristics, risk adjustment, and any expert panel comments. Data collection tools, with practical collection commentary, will be designed for each manual in order to capture each preliminary QI during Phase 3 (field-testing). To ensure a balance between comprehensive testing of QIs and timely data collection in Phase 3, those data items that assist in scoring the majority of QIs will be included. Data items deemed ‘not feasible’ will be removed and this will lead to some QIs not being able to be scored. All excluded indicators from the expert panel will be documented in a separate manual, with the reasons for exclusion clearly recorded.

### Phase 3: evaluate the preliminary musculoskeletal QI set

*(This phase commenced in 2016 and is ongoing).*

#### Aim

The aim of this phase of the project is to field-test the preliminary QI set. The validity, reliability, feasibility and usefulness of each of the preliminary QIs will be tested by collecting data from a representative sample of patients with musculoskeletal injuries presenting to a representative number of EDs throughout the state. The testing will conclude with complex analytical processing of the data that involves risk adjustment.

#### Design

The field-testing phase of this project will be a multi-centre prospective observational cohort study. Data to score all QIs will be collected across eight EDs in Queensland, Australia, in order to test the feasibility and validity of the preliminary set, and is based on previous QI development methodology [38, 39].

Patient recruitment and data collection is expected to be conducted for 8 weeks across each of the eight sites. It is anticipated that total recruitment across all sites will continue for 8–12 months, as each site will not recruit concurrently.

#### Participant groups

Site selection is based on case-mix, the ability to recruit adequate patient numbers, allied health models of care, and recognition of a balanced representation of district, metropolitan and tertiary centres. Table 1 outlines the profiles for the selected EDs based on data at the time of site selection.

**Table 1:** Site profiles

Site	ACEM role delineation [54]	Queensland clinical services capability framework [55]	Average musculoskeletal presentations/day 2013–14 (n)	ED physiotherapy staffing
A	Urban district	5	26	Primary and secondary contact
B	Major referral	6	32.13	Secondary contact
C	Major referral	6	34.8	Primary and secondary contact
D	Urban district	4	34.5	Secondary contact
E	Rural/Regional Base	3	17.1	Nil
F	Rural/Regional base	5	22.93	Nil
G	Major referral	6	36.5	Primary and secondary contact
H	Rural/Regional base	5	26.8	Primary and secondary contact

#### Sample size

Power analysis of the sample size is based on 60 participants at each of the sites, which is the minimum number for sufficient power to answer the research question. Recruitment of 80 participants at each of the eight sites, for a total of 640 participants, allows for completion of the study in view of drop-outs and incomplete data.

The adequacy of the sample size of at least 480 participants (60 at each site) is based on simulation methods and QI base rates. This sample size will have 77% power to detect reliability coefficients, where the estimated correlation among raters coefficient is >0.35 when the true value is 0.6, and the QI base rate is 50%. Using these parameters, we will be able to correctly classify the ED as ‘poor’ with an overall 83% accuracy. A classification analysis of ‘poor’ is the rate of patients flagging a QI greater than the observed 20^th^ percentile across facilities, when the true quality score for the facility is below the 20^th^ percentile. The c-statistic for this classification is 0.98, which is the proportion of facilities with their performance in the lowest 20%, that have observed quality scores in the lowest 20% [39]. The calculations to determine the minimum number required has been provided by A/Prof Rich Jones, of Harvard Medical School and has been used in an NHMRC funded grant on acute care quality indicators [38].

#### Participant inclusion and exclusion criteria

Inclusion criteria:

- adult patients presenting to the ED
- with a ‘presenting complaint’ ascertained from triage of potential musculoskeletal origin
- approached for consent within 2 h of triage if no initial treatment has commenced

Exclusion criteria:

- patients presenting with injuries involving high-velocity trauma, pregnancy, highly dependent on medical care and unable to give consent
- inadequate cognition required to provide informed consent
- not presenting with a primary complaint of a musculoskeletal origin
- non-English speaking without timely access to an interpreter

Participants will also be excluded if the Research Assistant (RA) was unable to approach them for consent because:

- they attended the ED outside the recruitment hours
- the patient was being attended by a staff member when the RA was available to recruit
- the RA was with another patient and could not recruit the patient within the required timeframe

Any recruited participant who returns to ED during the recruitment period will not be recruited a second time. Their return visit to the ED will be recorded as an event in the study.

#### Data collection

The data collection methods will include:

- *Prospective observational data collection:* A musculoskeletal questionnaire, which is based on a structured patient interview style [48, 49] will be administered to participants in the ED waiting room. This tool will collect information on the participants' demographics, health status, specific information related to their acute injury, and outcome measures, and will incorporate existing validated tools. The participant will then be observed by an RA throughout the entirety of their ED stay, where they will undergo usual ED assessment and management by the appropriate ED staff member. The RA will score against an extensive data collection tool as they observe the participant. This may involve some verbal interaction with the patient, such as asking for their pain score, but will predominantly involve silent scoring against a checklist of assessment and treatment items that were either performed or not performed by the treating clinician in ED.
- *Phone follow-up:* Follow-up phone calls at one week and six weeks post- ED presentation will conclude the prospective data collection. The phone follow-up will collect data on any adverse events following the ED episode, additional ED or hospital admissions, outcomes of any follow-up required (e.g. Fracture Clinic), pain scores and medication management following the ED episode, return to work and/or function, patient satisfaction, patient perception of clinical decision making and privacy.
- *Chart audit:* Each participant's medical record will be reviewed using a chart-abstraction tool, no sooner than 2 months post- ED presentation. This allows time for all relevant information to be filed in the chart or electronic medical record. The aim of the chart audit is to identify any disparities in recording between the patient’s care in ED, and documentation in the medical record. The data elements collected in the chart audit will be almost identical to those collected in the observational data collection tool and follow-up phone call tool. The chart audit is crucial to identify whether this method of data collection is a reliable way to score the QIs, as it more cost-effective than the prospective observational data collection.
- *Site visit:* A survey assessing environmental factors relating to clinical care will occur at each site. This will include data collection on structural processes such as equipment, staffing, layout, and policies and procedures relevant to musculoskeletal injuries within EDs.
- *Demographic data collection for consenting patients (Data extraction from EDIS):* Data concerning basic patient demographics and injury severity of consenting participants will be collected retrospectively from EDIS. This includes: age, gender, nationality, recognition of Aboriginal or Torres Strait Islander status, arrival mode, arrival day and time, type of visit, triage category, waiting time, discharge destination, and time spent in ED. Data collected from EDIS will be compared to the data collected during the observational data collection.
- *Demographic data collection for all musculoskeletal injuries (EDIS):* The State Data Custodians will be asked for data on all relevant International Classification of Diseases (ICD) codes (i.e. all musculoskeletal diagnosis groups) who presented to the ED in the 8-week block of recruitment. The data will be de-identified. Data points of interest are the same as the demographic data points in 5a. This report will give information on all patients with musculoskeletal injuries who attended that ED in the specific period of time. This data will be compared to the data from 5a, to assess whether the sample group of consenting patients is representative of all presentations.
- *Subsequent presentations data collection:* The State Data Custodians will be asked for data on the participants’ ED episode and any subsequent and additional hospital interactions in the 28 days post ED presentation. ICD codes, Diagnosis-Related Groups (DRG) for classifications, length of stay of participants, and discharge destination for subsequent presentations will be requested.

#### Research staff

All RAs and hospital staff will be blinded to individual QIs. The data collection forms will be designed to not reveal QI definitions.

Site RAs will be employed to conduct the prospective data collection and site audits at each of the eight sites. These RAs will conduct the follow-up phone calls during the recruitment period at that site. When recruitment has concluded, experienced staff at QEII Jubilee Hospital Physiotherapy Department will carry out any remaining phone calls. Different research staff will be employed to complete the chart audits, and they will be blinded to the prospective data collection results. A data collection manual designed specifically for the RAs will be developed and issued to all RAs, detailing their role and the data collection protocol, including any definitions or rules for certain data elements.

All RAs will be provided with comprehensive training prior to the field study commencing. Each RA conducting the site data collection (observational data collection, follow-up phone calls, and site audits) will receive individual training within their ED by a member of the research team at the beginning of their 8-week block of data collection. Separate training of chart auditors will include training on the abstraction protocol, supervised practice charts, independent chart review using the chart abstraction tool, followed by comparison with the trainer. Co-review of five charts will take place during training until excellent inter-rater reliability between chart auditors is achieved (kappa >0.7 and percentage agreement of all variables >90%). Definitions and rules will be created via joint collaboration between the trainer and the chart auditors for each individual variable that did not meet agreement, and subsequently added to the data collection manual. The chart auditors will then conduct an additional three chart audits, and variables with low agreement (≤60% agreement) from the first five charts will be checked for reliability by the trainer. Any remaining disagreement will be further discussed and variables reworded collaboratively to ensure consistency with scoring, again being added to the RA data collection manual.

#### Participant recruitment

Participants will be recruited by the site RA who will scan EDIS for patients with a presenting complaint that meets the inclusion criteria of the study. Eligible patients will be approached in consecutive order, after they have been triaged and registered, and prior to any treatment commencing. The site RA will explain the purpose of the study, outline the range of questions that will be asked and the anticipated duration of the patient’s involvement in the study. They will be supplied with a Patient Information Sheet. Patients who agree to participate in the study will be asked for written consent prior to commencing data collection.

If a patient who is approached to participate in the study does not wish to participate, all contact between the site RA and the patient will cease at that time. The patient’s decision not to participate will not affect their routine treatment or their relationship with those treating them.

Prior to each patient being approached for recruitment, they will be assigned a unique research identification number which de-identifies the patient, and this will be stored in a recruitment database. Non-consenting patients will be recorded in the database against this ID number as ‘non-consenting’. No chart review or follow-up phone calls will occur for these patients. Non-consenting patients may be re-approached for inclusion in the study if they return to ED, as there will be no record of their identification to check for any prior presentations to ED.

If a patient becomes ineligible or is excluded, basic general demographic information will be recorded as per above, and the reason for ineligibility noted on the data collection form.

Participation is voluntary and patients can withdraw at any time. If a participant chooses to withdraw they can either stop data collection (but have the data that has already been collected remain in the study), or choose to exclude their data from analysis in the study. If a patient chooses to withdraw, they can provide verbal or written advice to the RA, or fax/phone the request to the research team. Withdrawal forms will be available from the RA. The options to withdraw (cease data collection) or withdraw (exclude data) will be documented by the research team in the comments box on the withdrawal form.

#### Data management

A password-protected Microsoft Excel tracking database will be formulated to allow for recruitment rates at each site to be regularly monitored. Participant data anonymity will be preserved by being assigned a unique identifying number. This number will be used in the study database rather than any personally identifying information.

Data from the data collection tools will be entered into specifically designed electronic databases, linking data items from each tool to allow for assessment and analysis. To allow for data from different collection points to be matched and merged, the data will need to remain potentially identifiable by using a unique identifying number. As data collection for a single patient will occur on different tools, at different times, it is imperative that the process allows for all data to be matched to that same patient. The data will then be cleaned to improve the accuracy of data collected and entered. Once all the data has matched, the dataset will be made de-identifiable. Participants will not be able to withdraw their data from the study after the data has been de-identified as there will be no linkage at this stage.

Integrity of data will also be preserved by adopting a standardised approach across the sites for research assistant training on use of the tools, data collection, data submission, and data cleaning. The research investigators and other invited key personnel will meet fortnightly to monitor the study’s progress.

#### Data analysis

Data will be entered into SPSS, where frequency and distribution statistics will be used to describe the data set in basic terms (e.g. mean age, number of females). Data analysis will occur in 4 steps:

1. The data will be cleaned to check for data integrity and to ensure reliability
2. General descriptive data will be obtained from the data set using frequencies, standard deviations and means, to gain an understanding of the data sample
3. Each QI will be defined by data elements. Scoring of each QI will occur by running data analysis to score individual indicators within the data set
4. A unit-level analysis will evaluate the reliability and validity of the QIs using a multiple bootstrapped split-half correlation of patient samples and time-to-time correlations of repeated QI scores.

Based on the results of the field study, any relevant additional data analysis required for interpretation of the research findings will be run.

### Phase 4: establish final musculoskeletal QI set

*(This phase will commence in 2018).*

#### Aim

The aim of this phase is to reconvene the expert panel to review the preliminary QI set along with the data from the field-study (Phase 3). The expert panel, containing the same members from the first meeting in Phase 2, will comment on, refine or exclude indicators, based on an individual indicator’s capacity to reflect quality of care (lack of variability or feasibility), prior to the voting round. The voting rounds will result in the establishment of a final QI suite, encompassing structure, process and outcome measures of quality of care.

#### Design

The final expert panel meeting will again occur at a face-to-face meeting over 2 days, where they will focus on QI face and content validity, the clinical significance of each QI, and the ability of ED staff to impact on the indicators. Feasibility will not be considered a priority when voting, as this factor is highly variable depending on the priorities and resources of the data collector. It also changes as the data collection procedures change over time. For example, a QI that is considered challenging to collect today, but clinically important, can become very feasible tomorrow, if legislation changed and it became mandatory.

Pre-defined methodology for identifying a valid indicator has been used in other studies for the development of QIs [38, 39, 44]. The list includes:

- measurability: the QI is able to measure that which it pertains to measure
- control: the capacity to change the outcome is within the control of the ED
- change: there is sufficient scope to change the QI. Ie. It currently triggers between 5 and 95% of the sample
- evidence: there is evidence to show that there are interventions which change the outcome.

#### Data analysis

During the final expert panel meeting, a summary document will be presented to the panel describing the QI name, denominator, numerator and exclusion criteria. The summary will include relevant evidence, and results of the field-study data including prevalence of the trigger rates and percentage scores. The panel will be asked to consider each of the criteria above and vote on the validity of the QI at the end of the panel meeting.

The voting process is the RAND/UCLA appropriateness method in which voters score each QI from 1 to 9 in two rounds [50, 51]. The QI is only declared valid after it passes a series of decision rules which are decided prior. This is not a ‘majority rules’ process where a 50% majority passes the QI. The QI must be approved by the group to the extent where there is consensus. For each QI, the following will be calculated to determine consensus: the 30^th^ and 70^th^ percentile, the Interpercentile Range Adjusted for Symmetry (IPRAS), the Interpercentile Range (IPR), the Interpercentile Range Central Point (IPRCP), the Assymetry Index (AI), and the median [50]. Where there is ‘no consensus’ for a particular QI, then the QI is excluded. For each QI, if the IPRAS is greater than the IPR, there is agreement and if the median is in the range 7–9, there is consensus. If, in a particular domain, there is ‘no consensus’ but there are ‘undecided’ QIs (i.e. a wider range of opinions across the panel), the median is in the range 4–6, and the IPRAS is greater than the IPR, then the QI with the highest median may be included in the set.

A second voting round will occur after the second panel meeting. Each expert panel member will receive a summary of the expert panel discussion relevant to each QI, results from the first round of voting, and will be asked to vote again. If the first round of voting indicates a wide variability in the panel for a particular indicator, a teleconference will be held to discuss the definition to ensure confusion is eliminated as a cause for diversity. Only the second round of voting will be used to finalise the suite of QIs.

## Discussion

The establishment of a set of evidence-based valid and feasible QIs that reflect true levels of quality of care, will provide opportunities in improving the delivery of health service to this population in EDs. The QI set will allow for best practice to be applied and benchmarked across a variety of EDs. The results of this research will be timely given the rising number of musculoskeletal presentations to EDs each year and the increased pressures of meeting time performance targets.

The research protocol consists of development and testing phases, for structure, process and outcome musculoskeletal QIs [42]. No single indicator can encompass all dimensions of quality, thus a balanced set of QIs is required [52]. The efficient development of high quality QIs must utilise rigorous, approved and evidence-based methodology [53]. This research follows a previously published protocol to create valid QIs, which are characteristically specific, meaningful, comparable, amenable to improvement, and efficiently measurable [38, 39].

The final QI suite will have defined elements, with a definitive method of scoring, allowing for research consistency in the future. This will be an immediate benefit and resource to the field of emergency medicine research, particularly for hospitals wishing to complete an audit or improvement activities, as the QIs will be a cost-effective measurement process where the focus can be on developing appropriate interventions rather than designing measures and targets.

These findings will contribute significantly to clinical research in the field of quality emergency health care, where current literature surrounding suites of indicators for specific conditions is limited to few conditions and non-existent in the field of musculoskeletal injury management.

## Abbreviations

ACEM: Australasian College for Emergency Medicine
AI: Assymetry index
DRG: Diagnosis-related group
ED: Emergency Department
EDIS: Emergency Department Information System
ICD: International Classification of Diseases
IOM: Institute of Medicine
IPR: Interpercentile range
IPRAS: Interpercentile range adjusted for symmetry
IPRCP: Interpercentile range central point
NHMRC: National Health and Medical Research Council
NQF: National Quality Forum
QI(s): Quality indicator(s)
RA(s): Research assistant(s)
SPSS: Statistical Package for the Social Science
UK: United Kingdom
USA: United States of America
